# Correction of a Severely Rotated Maxillary Incisor by Elastics in Mixed Dentition Complicated by a Mesiodens

**DOI:** 10.5005/jp-journals-10005-1320

**Published:** 2015-09-11

**Authors:** Mohsin Sidiq, Asif Yousuf, Manohar Bhat, Rajesh Sharma, Neha Bhargava, Shravani Ganta

**Affiliations:** Registrar, Department of Pedodontics, Government Dental College Srinagar, Jammu and Kashmir, India; Postgraduate, Department of Public Health Dentistry, Jaipur Dental College Jaipur, Rajasthan, India; Professor and Head, Department of Pedodontics and Preventive Dentistry, Jaipur Dental College, Jaipur, Rajasthan, India; Professor, Department of Pedodontics and Preventive Dentistry, Jaipur Dental College, Jaipur, Rajasthan, India; Senior Lecturer, Department of Pedodontics and Preventive Dentistry Rajasthan Dental College, Jaipur, Rajasthan, India; Postgraduate, Department of Public Health Dentistry, Jaipur Dental College Jaipur, Rajasthan, India

**Keywords:** Central incisor, Dental cross bite, Mixed dentition, Severe rotated tooth.

## Abstract

The aim of this case study was to report a potentially convenient approach instead of a conventional orthodontic procedure for correcting severe rotation of anterior tooth of an 11-year-old Indian boy, with a mixed dentition class I malocclusion. The child reported seeking treatment for severely rotated upper right central incisor with mesiodens and a single tooth crossbite. The supernumerary tooth was first extracted and bondable buttons were placed on the rotated tooth, an appliance composed of a removable plate with Adam’s clasp with distal extension and a loop for engagement of elastics was delivered. Circumferential supracrestal fibrotomy was performed on the corrected derotated tooth. Then, Hawley’s appliance with a z-spring and posterior bite plane was fabricated and placed for correction of crossbite. Thus, this removable appliance can be a simplified and a cost-effective treatment alternative for derotation of anterior tooth, especially during the mixed dentition period.

**How to cite this article:** Sidiq M, Yousuf A, Bhat M, Sharma R, Bhargava N, Ganta S. Correction of a Severely Rotated Maxillary Incisor by Elastics in Mixed Dentition Complicated by a Mesiodens. Int J Clin Pediatr Dent 2015;8(3):234-238.

## INTRODUCTION

Tooth rotation is defined as noticeable mesiolingual or distolingual intra-alveolar displacement of the tooth around its longitudinal axis. The prevalence of tooth rotation is 2.1 to 5.1% in the untreated population.^1^ Rotation of teeth can result from number of factors like— space availability for tooth alignment, tooth eruption order, and functional influences exerted by the tongue and lips, consonant with a multifactorial model in the origin of tooth malpositions. Supernumerary teeth are found most commonly in maxilla, of which mesiodens is the commonest anomaly.^2^ It can also cause tooth rotation, delay or prevent eruption of central incisors; cause ectopic eruption, displacement or rotation of a central incisor and labially displaces incisors.^3^ Other, less frequent problems include—root resorption of adjacent teeth, dentigerous cyst formation, nasal eruption of supernumerary teeth, dilacerations of the developing roots and loss of tooth vitality.^4^ Tooth rotation poses greater difficulty for correction, if the tooth in rotation is compounded with adjacent tooth malposition and inadequate space in the arch.^5^ The later the extraction of the mesiodens, the greater the chance that the permanent tooth either will not spontaneously erupt, or will be malaligned when it does erupt.^6^ In a dental arch with crowding, rotations are often present; but in cases of excess space also, rotations might occur.^7^ The purpose of presenting this case report was to introduce an alternative approach to conventional orthodontic procedure by introducing a removable appliance, to overcome the disadvantages of the conventional fixed appliance.

## CASE REPORT

An 11-year-old male patient reported to department of pedodontics, Jaipur Dental College with the complaint of irregularly positioned upper right front tooth (Figs 1A to D). The extraoral examination of the child revealed mild convex profile, and in frontal view he was mesoproscopic, had a symmetric face and competent lips at rest (Fig. 1A). The child’s medical history was non-contributory. The intraoral examination revealed mixed dentition in both the arches with class I molar relation. The maxillary right central incisor was mesiolabially rotated with a mesiodens present, and maxillary right lateral incisor was palatally erupted, and in crossbite (Figs 1B and C). Oral hygiene was fair with mild gingivitis. The parents were informed about the malocclusion and a written consent to proceed with the treatment, aimed at de-rotation followed by correction of the crossbite, was obtained.

**Figs 1A to D:**
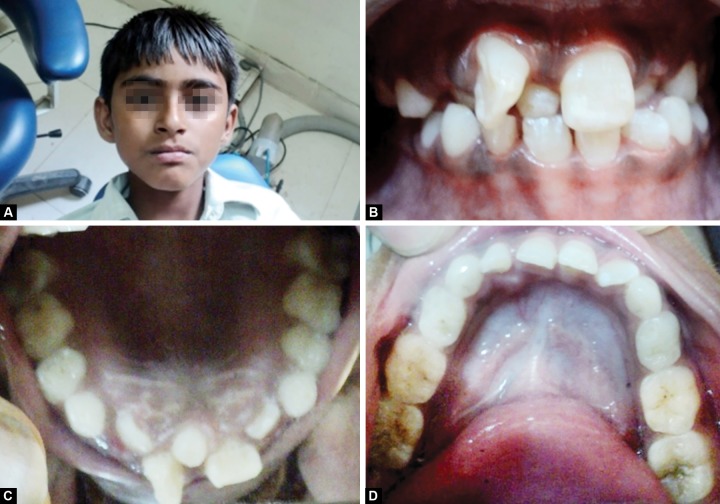
Extraoral and intraoral examination

After extraction of the mesiodens, total space analysis revealed adequate space for the mesiodistal alignment of rotated central incisor. After 2 weeks of extraction of mesiodens, bondable buttons were placed on the labial and palatal surfaces of upper right central incisor (Fig. 2A). Fabrication of removable appliance, made of an acrylic base plate with modified Adam’s clasp with distal extension in relation to upper left central incisor and a loop for engaging elastics (Fig. 2B), was fabricated near the deciduous upper right canine. Elastics were placed between palatal bondable button and the distal extension of adams clasp, and also between the labial bondable button and the loop (Figs 2C and D). After 4 months of follow-up, bondable buttons were removed (Figs 2E and F); and pericision/circumferential supracrestal fibrotomy was performed (Fig. 3A). For overcorrection of the rotated tooth, a Hawley’s appliance was fabricated with a z-spring on upper right lateral incisor with posterior bite plane for correction of cross bite (Figs 3B to D). At the end of the treatment, fixed palatal retainer was placed to prevent relapse (Figs 3E and F).

## DISCUSSION

One of the most common causes of severe rotation of upper incisors is the presence of supernumerary teeth. The associated complications include—lack of eruption of permanent teeth, deviation from the eruption path, rotations and root resorption.^8^ The archetypal treatment for teeth rotations is a fixed ‘2 × 4’ appliance in the mixed dentition (two bands on first molars and four bonded brackets on incisors). Simple fixed appliances used in the mixed dentition can be quite complex to use appropriately, and used only after complete eruption of at least permanent first molars and incisors.^9^ Another disadvantage of the fixed appliance is difficulty in the maintenance of oral hygiene that can lead to decalcification of banded and bonded teeth.^10^

The second alternative treatment for derotation in some particular situations is a removable appliance with a labial bow and a palatal spring like z-spring, which provides the moment to derotate the tooth. In this appliance, the reactive forces are less; therefore, there is no particular problem in anchorage. In addition, if the palatal surface of the rotated tooth is positioned along the dental arch, a removable appliance with labial bow and base plate can be used, and the contact of tooth with acrylic base plate at the second moment is adequate.^11^ One of the disadvantages of this method is that it may be indicated only in the case of maxillary central incisor, and probably can only correct mild rotations less than 45°. Furthermore, rotations have very high risk of relapse and because patient compliance is needed in the removable appliance, relapse even in the treatment phase is more likely. Another disadvantage of this appliance is its need for accurate adjustment of the labial bow, palatal spring and acrylic of the base plate.

**Figs 2A to F:**
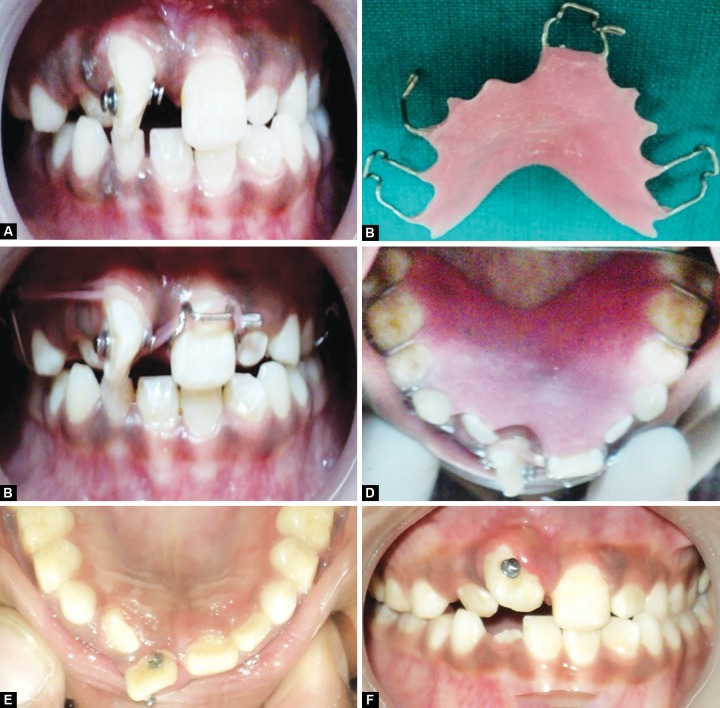
Placing bondable buttons, removable appliance with modified Adam’s clasp with distal extension and placement of elastics

With the use of removable appliance in the whip device, a good anchorage unit is provided from the entire palate and the maxillary dentition, and thus can be suggested for correcting a severely rotated central incisor in the mixed dentition. Problems that may be encountered during treatment are debonding of the bracket and distortion of the spring. However, these problems can be minimized through satisfactory compliance. Other undesirable effects are extrusion and slight labial tipping of upper incisors during treatment.^12^ Furthermore, the whip spring can wound the mucosa if not adjusted carefully.^13^

Because of the limitations of above discussed methods, in this case, we introduced a removable appliance with fixed bondable buttons on the rotated tooth; over which the elastics were engaged for treatment of the incisor rotation, to which a force to rotate a tooth can be applied by the elastics. This force does not have harmful side effects on tooth development. It has been suggested that since root shortening due to apical resorption is one of the most serious side effects of orthodontic treatment, it appears advisable to initiate orthodontic correction of the incisors at a young age during mixed dentition, in an introductory phase of treatment.^14^ Before derota-tion is undertaken, it is important that sufficient space is available to accommodate the tooth in alignment. Our technique has several advantages for use in the mixed dentition. This appliance solves the problem in mixed dentition, relatively in a short duration. The management of anchorage is less critical and the force system is relatively simple. The appliance is removable and better oral hygiene management is possible. There are no lacerations of mucosa due to absence of active wire component. The main problem of this technique is decalcification as a result of plaque accumulation around the buttons and the debonding of the buttons, but satisfactory compliance can minimize this problem. Removable appliances may also be associated with gingival inflammation, particularly of the palatal tissues, in the presence of poor oral hygiene. Since derotated teeth are prone to relapse, they must be overcorrected and retained.

**Figs 3A to F:**
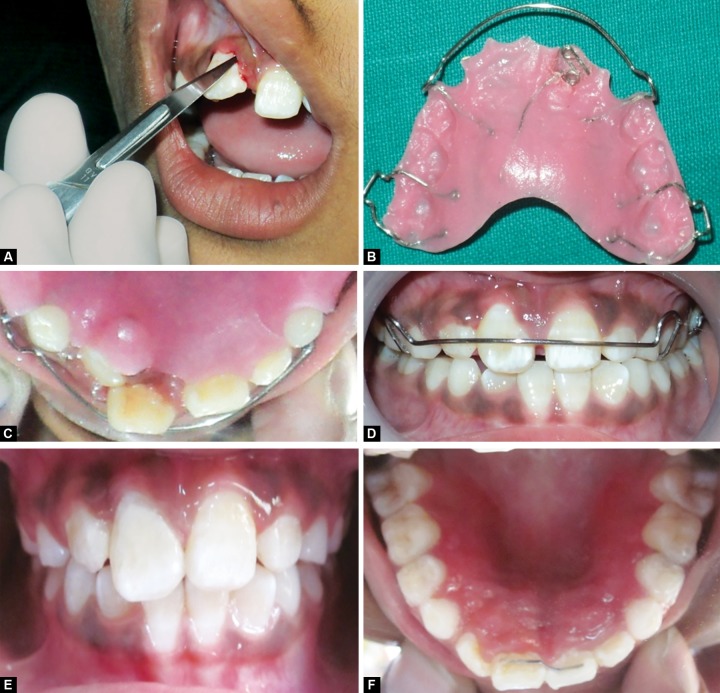
Circumferential supracrestal fibrotomy done, Hawley’s appliance with a z-spring and posterior bite plane and placement of fixed palatal retainer

## CONCLUSION

This removable appliance offers a simplified and a cost effective treatment alternative employed for successful derotation of anterior tooth especially during the mixed dentition period. But ideal case selection, patient’s cooperation and compliance is mandatory for desired results.
